# The Influence of Elevated CO_2_ on Volatile Emissions, Photosynthetic Characteristics, and Pigment Content in *Brassicaceae* Plants Species and Varieties

**DOI:** 10.3390/plants11070973

**Published:** 2022-04-02

**Authors:** Andreea Lupitu, Cristian Moisa, Simona Gavrilaş, Mihaela Dochia, Dorina Chambre, Virgiliu Ciutină, Dana Maria Copolovici, Lucian Copolovici

**Affiliations:** Faculty of Food Engineering, Tourism and Environmental Protection, Institute for Research, Development and Innovation in Technical and Natural Sciences, Aurel Vlaicu University of Arad, Elena Drăgoi St., No. 2, 310330 Arad, Romania; pag.andreea@yahoo.com (A.L.); moisa.cristian@yahoo.com (C.M.); simona2213@yahoo.com (S.G.); dochiamihaela@yahoo.com (M.D.); dorinachambree@yahoo.com (D.C.); virgilciutina@yahoo.com (V.C.); dana.copolovici@uav.ro (D.M.C.)

**Keywords:** green leaf volatiles, induced emissions, monoterpene emission, photosynthesis, quantitative responses, volatile organic compounds

## Abstract

Climate change will determine a sharp increase in carbon dioxide in the following years. To study the influence of elevated carbon dioxide on plants, we grew 13 different species and varieties from the *Brassicaceae* family at three carbon dioxide concentrations: 400, 800, and 1200 ppmv. The photosynthetic parameters (assimilation rate and stomatal conductance to water vapor) increase for all species. The emission of monoterpenes increases for plants grown at elevated carbon dioxide while the total polyphenols and flavonoids content decrease. The chlorophyll content is affected only for some species (such as *Lipidium sativum*), while the β-carotene concentrations in the leaves were not affected by carbon dioxide.

## 1. Introduction

Carbon dioxide is the main greenhouse gas emitted into the atmosphere due to human activities. Global CO_2_ concentration has increased more than ever in the last 20 years as the carbon dioxide amount has grown by 43.5 ppmv, increasing 12 percent. The actual concentration of carbon dioxide will exceed 420 ppmv at the end of 2021. The projected CO_2_ concentrations in 2100, under a range of emissions scenarios developed for The Intergovernmental Panel on Climate Change (IPCC), vary from 500 ppmv to 1200 ppmv [1]. The critical impacts of projected climate change on plants are considered inevitable [2]. The increase in carbon dioxide emission could lead to more climate change, including different episodes of extreme heat, droughts, or flooding that stress terrestrial vegetation [3]. Plants can respond to rising atmospheric CO_2_ concentrations by increasing the water-use efficiency and photosynthetic rates [4,5,6]. Free-air CO_2_ enrichment (FACE) experiments, which use plants grown in open-air environments enriched with carbon dioxide, have been shown to increase the yield of food crops, even compared to chamber experiments [7].

Regarding photosynthetic parameters, increasing carbon dioxide determines an increase in photosynthesis for C3 plants [8,9]. On the other hand, different studies have shown elevated carbon dioxide downregulation of photosynthetic capacity [10]. Such limitation has been found for *Quercus ilex* L. leaves and has probably been due to the low capacity for ribulose-1,5-bisphosphate regeneration [11]. The net photosynthetic rate dropped for *Glycine max* (L.) plants grown at elevated carbon dioxide levels due to stomatal traits and mesophyll tissue size changes [12]. 

The emission of volatile organic compounds (VOC) from plants grown at high carbon dioxide is enhanced compared with plants grown at the actual CO_2_ concentration (see [13] for a review). For example, the isoprene emission increases in *Phragmites australis*, *Platanus* × *acerfolia*, and *Populas nigra* × *maximowiczii* NM6 when grown in high carbon dioxide [14]. The abundance and diversity of plant volatile organic compounds emission from *Hordeum vulgare* L. seedlings, including aromatics, terpenes, and green leaves volatiles, were also changed by elevated CO_2_ compared with the actual carbon dioxide concentration [15]. In contrast, foliar VOC concentrations are unaffected by the growth conditions (700 ppmv CO_2_ compared to 400 ppmv CO_2_) for *Artemisia annua* plants [16]. 

It has been found that elevated CO_2_ increases the foliar contents of polyphenols of tea seedlings but decreases in free amino acids and caffeine [17]. Carbon dioxide fertilization has not affected the total phenols determined in chickpea leaves [18]. Leaf polyphenol concentrations found in two Cerrado native species, *Baccharis dracunculifolia* and *B. platypoda*, grown at 400 ppmv and 800 ppmv carbon dioxide, have not been statistically significant [19]. 

The concentration of chlorophylls in the leaves of plants grown at elevated carbon dioxide decreases despite the total phenols concentration. The rate of decline in the chlorophyll contents of *Oryza sativa* L. leaves was faster in plants grown under high carbon dioxide, mainly in the later growth period [20]. Exposing tomato leaves to various CO_2_ treatments revealed a decrease in chlorophyll *a* and *b* [21]. In a recent study with terrestrial plants, it has been shown that chlorophyll fluorescence decreases with an increase in carbon dioxide concentration [22]. In contrast, the total chlorophyll increased in chickpea grown at 700 ppmv CO_2_ compared to the ambient conditions at the flowering stage. Such behavior could be explained by symbiotic nitrogen fixation, which can fulfill its N requirement through plant N uptake, accelerated under elevated atmospheric CO_2_ [23]. An increase in chlorophyll content with 33% has been found for pak choi (*Brassica rapa* ssp. *chinensis*) plants grown at 800 ppmv carbon dioxide compared to the control [24]. It was also found that elevated carbon dioxide could increase the concentrations of photosynthetic pigments (chlorophylls and carotenoids) in different sorghum genotypes [25]. 

The synthesis of total flavonoids and monomers in the leaves of *Robinia pseudoacacia* L. seedlings is positively affected by elevated carbon dioxide, especially for older plants [26]. Elevated CO_2_ concentration can cause higher chlorogenic acid accumulation in *Lactuca sativa* L. plants [27], while in barley and maize, high CO_2_ decreases the total flavonoids and anthocyanins [28]. The summarized data on the effects of elevated carbon dioxide regarding different plant characteristics are presented in Table 1. 

The plants from the *Brassicaceae* family have economic and agricultural importance as they represent an important dietary source of glucosinolates, vitamins, polyphenols, and minerals. The data regarding the influence of elevated carbon dioxide on *Brassicaceae* plants’ characteristics are scarce. An early study showed that two *Brassica rapa* cultivars grows at 550 ppmv CO_2_ showed increased crop productivity, even with the high-end soil nitrogen [34]. An increase in chlorophyll content of 33% has been found for pak choi (*Brassica rapa* ssp. *chinensis*) plants grown at 800 ppmv carbon dioxide compared to the control [24]. In *Brassica rapa* plants that grew at 700 ppmv carbon dioxide, the higher phenolic compounds increased the resistance to herbivore stress [35]. 

To evaluate the impact of two elevated carbon dioxide concentrations on the different plants, we grew plants at 800 and 1200 ppmv, respectively. Those concentrations could be achieved in the ambient atmosphere in the year 2100, according to the IPPC models [1]. To the authors’ best knowledge, this is the first study that determines the plants’ response regarding secondary and primary metabolites for plants grown at a higher than 1000 ppmv carbon dioxide concentration. This study also aims to determine the photosynthesis parameters, pigments, and secondary metabolites of 13 different *Brassicaceae* species and/or varieties to characterize plant signaling at elevated carbon dioxide. 

## 2. Results

### 2.1. The Influence of Elevated Carbon Dioxide on Photosynthetic Parameters

**Figure 1:**
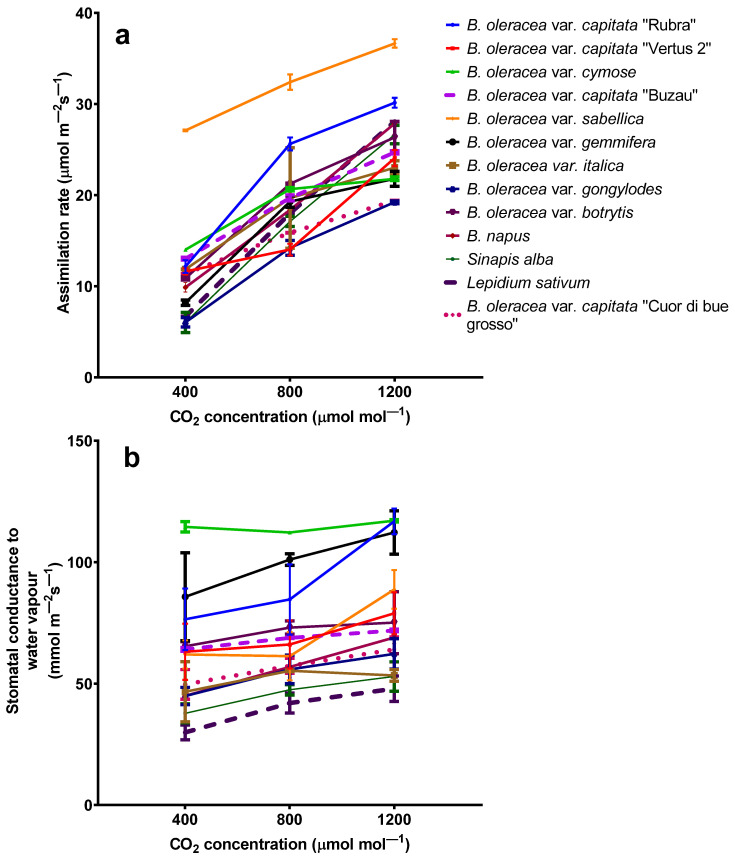
The assimilation rate (**a**) and stomatal conductance to water vapor (**b**) from *Brassicaceae* plants grown at three carbon dioxide concentrations. The values are averages of three independent measurements.

The elevated carbon dioxide upregulated the assimilation rates for all *Brassicaceae* plants. The highest assimilation rates for plants grown at 1200 ppmv were found in Kale cabbage (*Brassica oleracea* var. *sabellica*) at a level of 36.66 ± 0.46 µmol m^−2^ s^−1^, while the lowest assimilation rate was found for kohlrabi (*B. oleracea* var. *gongylodes*) (Figure 1a). Nonetheless, there are significant differences (one-way ANOVA followed by Tukey’s multiple comparisons *post hoc* test, *p* < 0.05) between the assimilation rates for all plants species grown at 400, 800, and 1200 ppmv carbon dioxide, despite the differences in the trend. For example, the most negligible differences in assimilation rates for plants grown at 400 ppmv CO_2_ compared with the one that was grown at 1200 ppmv CO_2_ was for *B. oleracea* var. *cymose* (14.01 ± 0.19 µmol m^−2^ s^−1^ and 21.79 ± 0.20 µmol m^−2^ s^−1^, respectively), while the greater values were found for *Lepidium sativum* (6.69 ± 0.21 µmol m^−2^ s^−1^ and 28.08 ± 0.18 µmol m^−2^ s^−1^, respectively). 

The stomatal conductance to water vapor values is not statistically different (one-way ANOVA followed by Tukey’s multiple comparisons *post hoc* test, *p* > 0.05) for plants grown at 400 ppmv and 800 ppmv for all *Brassica oleracea* varieties (Figure 1b). At the same time, there are statistical differences for *Brassica napus, Sinapis alba,* and *Lepidium sativum*. In contrast, there are statistical differences (one-way ANOVA followed by Tukey’s multiple comparisons *post hoc* test, *p* < 0.05) between the stomatal conductance to water vapor for plants grown at 1200 ppmv CO_2_ and those grown at 400 and 800 ppmv CO_2_. The highest stomatal conductance to water vapor was found for red cabbage (116 ± 5 mmol m^−2^ s^−1^), which increased at 1200 ppmv carbon dioxide, while the smallest was registered for cress (30 ± 3 mmol m^−2^ s^−1^).

### 2.2. The Emission of Volatile Organic Compounds from Plants under Elevated Carbon Dioxide

**Figure 2:**
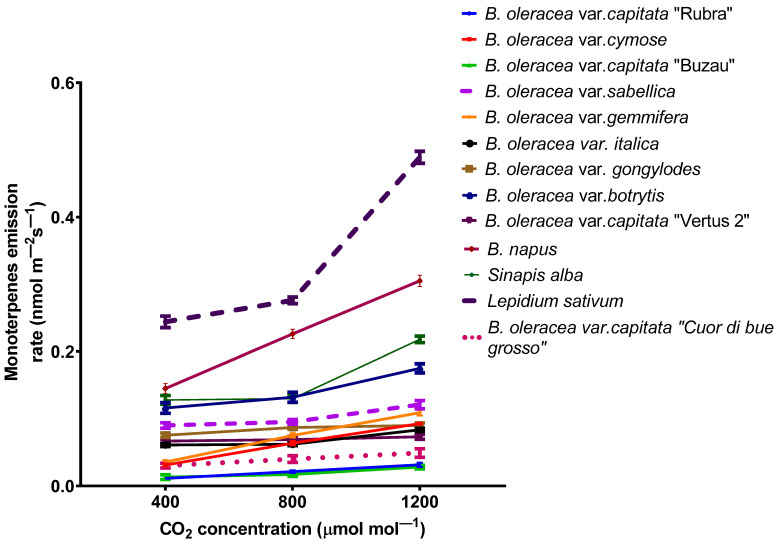
The emission rate of monoterpenes from *Brassicaceae* plants grown at three carbon dioxide concentrations. The values are averages of three independent measurements.

Among the volatiles, six monoterpenes (camphene, β-pinene, 3-carene, D-limonene, *para*-cymene, and γ-terpinene) were found in the emission of all 13 *Brassicaceae* plants. The total emission from all different species are pretty low but is increasing for plants grown at a high carbon dioxide concentration (Figure 2). The emission of terpenes was most abundant in cress, rapes, and white mustard, whereas the lowest levels were found in red and white cabbages. 

There is only one plant variety (*B. oleracea* var. *capitata* “Vertus 2”) with no significant emission differences between all three growing conditions (one-way ANOVA followed by Tukey’s multiple comparisons *post hoc* test, *p* > 1). In contrast, in all other 12 *Brassicacea* varieties, there is an increase in monoterpene emission, at least for plants that grow at 1200 ppmv carbon dioxide. On the other hand, for kale, broccoli, and cabbage (“Cuor di bue grosso”) the emission is not statistically different between plants that grow at 400 ppmv carbon dioxide and plants that grow at 800 ppmv carbon dioxide (one way ANOVA followed by Tukey’s multiple comparisons *post hoc* test, *p* < 0.05). 

### 2.3. The Influence of Elevated Carbon Dioxide on Chlorophylls and β-Carotene

**Figure 3:**
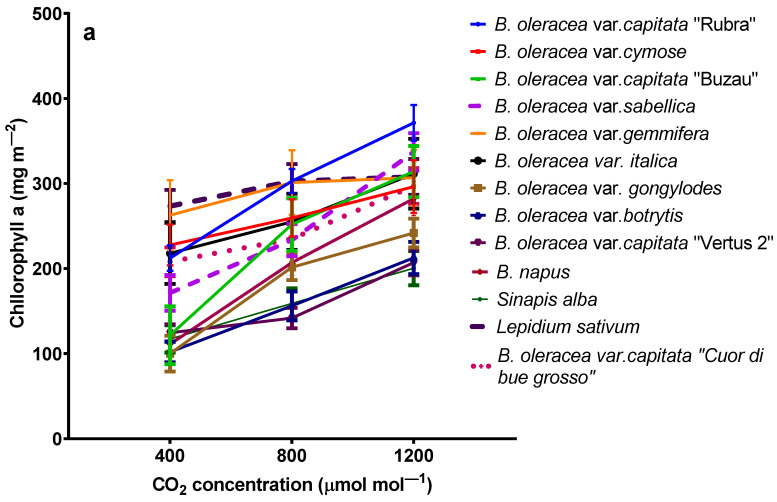

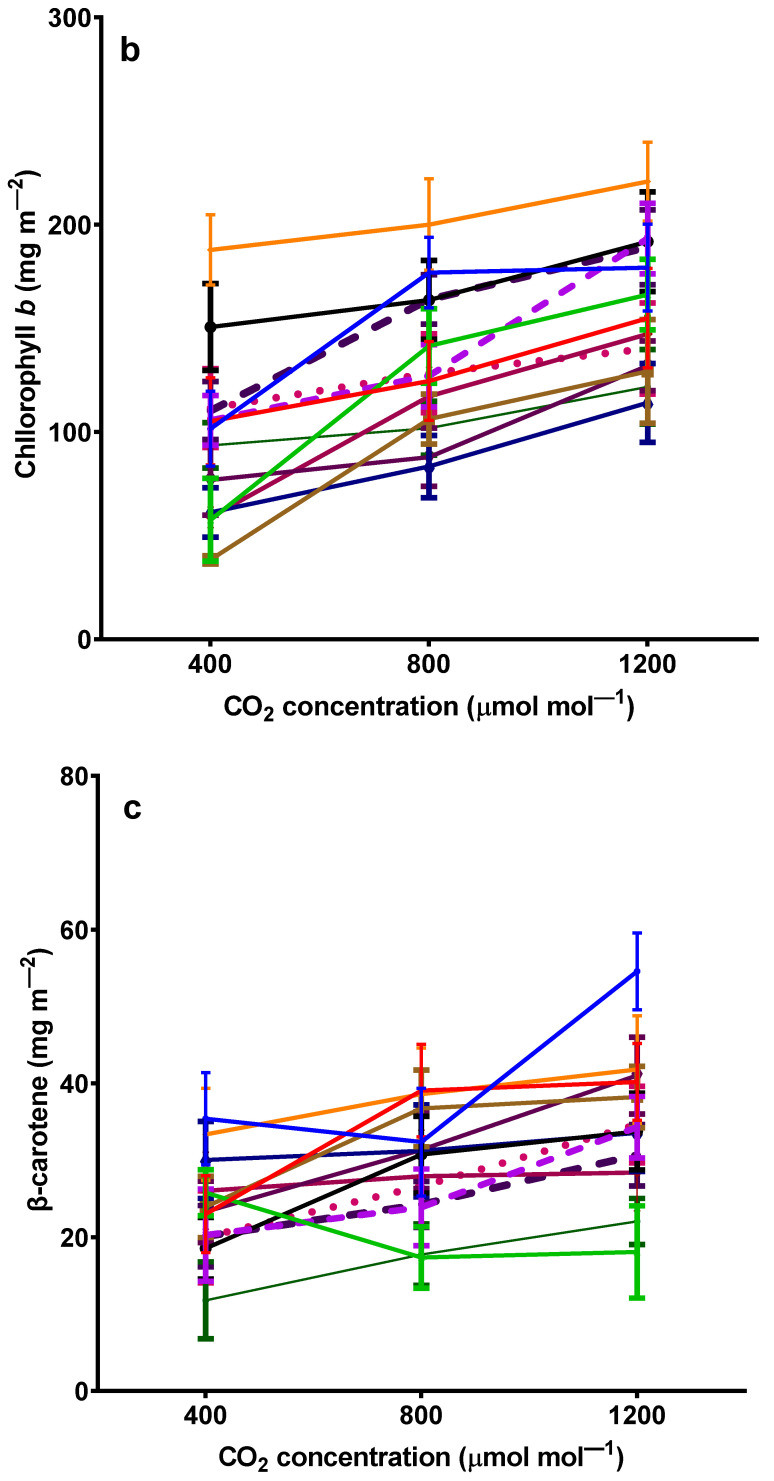
Chlorophyll *a* (**a**), Chlorophyll *b* (**b**) and β-carotene (**c**) concentrations from *Brassicaceae* plants grown at three carbon dioxide concentrations. The values are averages of three independent measurements.

The chlorophyll *a* concentration in leaves of all 13 *Brassicaceae* varieties increase with the increase in carbon dioxide concentrations (Figure 3a). The medium chlorophyll *a* concentrations in the leaves of all 13 plants were 173 ± 63 mg m^−2^ for plants grown at 400 ppmv CO_2_, 231 ± 56 mg m^−2^ for plants grown at 800 ppmv CO_2_, and 283 ± 21 mg m^−2^ for plants grown at 1200 ppmv CO_2_, respectively. The chlorophyll *a* concentration for plants grown at 400 ppmv CO_2_ varies from 100 ± 21 mg m^−2^ in *B. oleracea* var. *gongylodes* to 273 ± 19 mg m^−2^ in *Lipidium sativum*, while from plants grown at 1200 ppmv CO_2_ vary from 200 ± 20 mg m^−2^ in *Sinapis alba* to 371 ± 21 mg m^−2^ in *B. oleracea* var. *capitata* “Rubra”. 

The chlorophyll *b* concentrations generally increase for plants grown at high carbon dioxide concentrations, but it is not general for all varieties (Figure 3b). The medium chlorophyll *b* concentrations in the leaves of all 13 plants were 97 ± 40 mg m^−2^ for plants grown at 400 ppmv CO_2_, 132 ± 35 mg m^−2^ for plants grown at 800 ppmv CO_2_, and 160 ± 33 mg m^−2^ for plants grown at 1200 ppmv CO_2_, respectively.

The β-carotene concentration does not depend on the carbon dioxide growing conditions except for red cabbage (Figure 3c). 

The medium β-carotene concentrations in the leaves of all 13 plants were 24 ± 6 mg m^−2^ for plants grown at 400 ppmv CO_2_, 29 ± 7 mg m^−2^ for plants grown at 800 ppmv CO_2_, and 35 ± 9 mg m^−2^ for plants grown at 1200 ppmv CO_2_, respectively. There are only statistical differences between plants grown at 1200 ppmv and 400 ppmv CO_2_ (one-way ANOVA followed by Tukey’s multiple comparisons *post hoc* test, *p* < 0.01).

### 2.4. The Change in Total Phenol Concentration for Plants Grown at Different Carbon Dioxide Concentration

**Figure 4:**
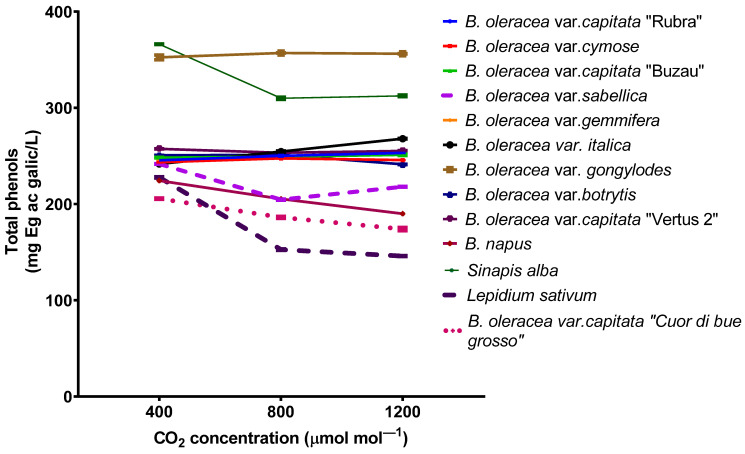
Total phenols concentration from *Brassicaceae* plants grown at three carbon dioxide concentrations. The values are averages of three independent measurements.

Elevated carbon dioxide does not affect the total phenols concentration for some varieties but decreases for others (Figure 4). The most pronounced decrease in total phenols concentration was found in *Lepidium sativum* plants (227 ± 7 mg Eg gallic acid/L for plants grown at 400 ppmv compared with 145 ± 5 mg Eg ac gallic/L for plants grown at 1200 ppmv).

### 2.5. The Influence of Elevated Carbon Dioxide on Flavonoids Content in the Leaves of Brasicacea Plants

**Figure 5:**
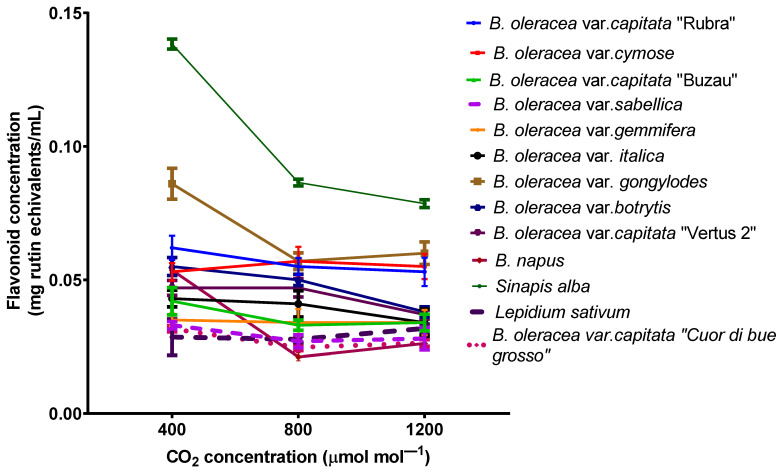
Total flavonoids content from *Brassicaceae* plants grown at three carbon dioxide concentrations. The values are averages of three independent measurements.

The total flavonoid contents in leaves generally decrease for plants grown at elevated carbon dioxide, but there is no clear trend (Figure 5). There are some species (such as *Sinapis alba*) in which the concentration decrease significantly (one way ANOVA followed by Tukey’s multiple comparisons *post hoc* test, *p* < 0.001) for plants grown at elevated carbon dioxide, while in others the concentration of flavonoids is not affected (as in *B. oleracea* var. *gemmifera*).

The medium total flavonoids concentrations in the leaves of all 13 plants were 0.054 ± 0.030 mg rutin equivalents/mL for plants grown at 400 ppmv CO_2_, 0.043 ± 0.018 mg rutin equivalents/mL for plants grown at 800 ppmv CO_2_, and 0.041 ± 0.0016 mg/mL for plants grown at 1200 ppmv CO_2_, respectively. There are no statistical differences between plants grown at different CO_2_ concentrations (one-way ANOVA followed by Tukey’s multiple comparisons *post hoc* test, *p* > 0.1).

### 2.6. Microscopic Analyses

**Figure 6:**
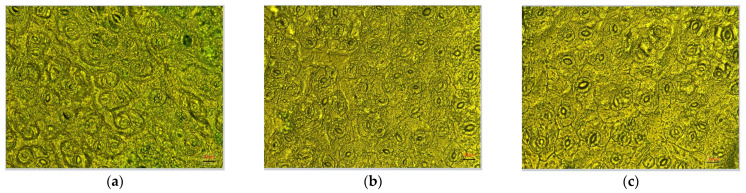

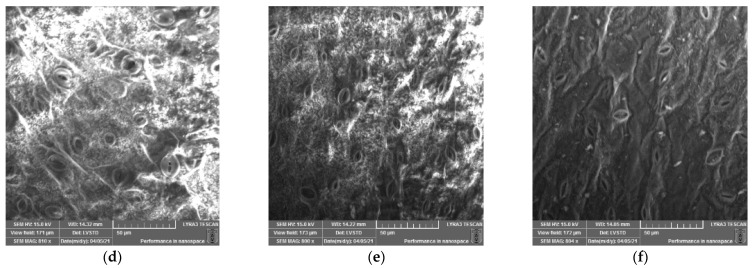
Microscopy images that present the stomatal pattern of leaf surfaces of *Brassica oleracea italic* recorded with an optical microscope ((**a**–**c**), with the scale bar 20 μm) and SEM micrographs ((**d**–**f**), magnification 800×) for leaf plants grown at 400 (**a**,**d**), 800 (**b**,**e**), and 1200 (**c**,**f**) ppmv CO_2_.

Stomatal characteristics did not differ between plants from different treatment groups (Figure 6). The stomata length among the three treatments are 26.37 ± 3.09 µm for 400 ppmv CO_2_, 24.35 ± 1.84 µm for 800 ppmv CO_2_, and 26.73 ± 1.71 µm for 1200 ppmv CO_2_, which are not statistically different among treatments (one way ANOVA followed by Tukey’s multiple comparisons *post hoc* test, *p* = 0.7356). 

The pore apertures of plants grown at lower concentrations of CO_2_ remained larger than those of other plant groups. Stoma pore length slightly decreased from 14.65 ± 2.88 µm at 400 ppmv CO_2_ to 13.57 ± 1.93 µm at 800 ppmv CO_2_, but only became statistically different at 1200 ppmv CO_2_ (8.01 ± 0.88 µm, one way ANOVA followed by Tukey’s multiple comparisons *post hoc* test, *p* < 0.05).

## 3. Discussion

As expected, assimilation rates increased with the carbon dioxide concentrations for all species. The same results have been obtained from extensive experiments (FACE) in which legumes showed a 21% increase in saturated assimilation rates with growth at elevated CO_2_ (see [29] for a review). Carbon dioxide is collected in the substomatal cavities, which could determine the carbon dioxide fixation due to the reaction with RuBP in the presence of the RuBisCo enzyme [36]. Moreover, it has been shown that increasing CO_2_ in all species determines the decreasing percentage of leaf nitrogen allocated to RuBisCo, which suggests the acclimation of photosynthesis to elevated carbon dioxide [37]. Indeed, many studies have shown that elevated carbon dioxide increases the assimilation rates for C3 plants but decreases their nitrogen content, which is essential for vegetables [38,39]. 

Generally, under elevated carbon dioxide, stomata close due to higher depolarization of the guard cells [40,41]. The stomata closer to elevated carbon dioxide are induced by enhancing anion channel activity in guard cells [42]. Despite other experiments (in which stomatal conductance decreased on average by 20% in high carbon dioxide [43]), the stomatal conductance to water vapor is not affected by mildly increasing the carbon dioxide concentration. Such behavior has been found for *Cajanus cajan* L. during vegetative and reproductive growth phases [44] and could be due to altered guard cell signaling patterns. In *Arabidopsis* mutants with impaired Ca^2+^ priming sensors and HT1 protein kinase, the increase in carbon dioxide provoking stomatal conductance increased [45]. In our experiment, the stomatal conductance increases significantly only for plants grown at the highest carbon dioxide concentrations (1200 ppmv), which agrees with the results from previous papers [44,45]. 

The emission of terpenes from plants could be done from specialized secretory organs such as resin ducts, glandular trichomes, oil cavities, or *de novo* biosynthesis [46,47]. The monoterpenes emitted from plants in the atmosphere participates in secondary aerosol formations and could be implicated in different photochemical reactions. Generally, the emission of terpenes is decreasing for plants grown at elevated carbon dioxide concentrations [48]. Contradictory results have been found for the emission of volatiles from *Brassicaceae*. On the one hand, it has been shown that the emission of terpenes from *Brassica oleracea* ssp. *capitata* decrease for plants grown at high carbon dioxide [49] and, on the other hand, the emission of different volatile organic compounds from *Brassica napus* ssp. *oleifera* increase for plants grown at elevated carbon dioxide [50]. In our experiment, the monoterpene emission increased significantly (as in *Lepidium sativium*). In contrast, elevated carbon dioxide does not affect the emission for other species, as in the case of *B. oleracea* var. *sabellica*. Such data suggest that the monoterpene emission capacity of plants grown at elevated carbon dioxide is not affected by carbon accumulation in the leaf tissues [51]. As one of the most abundant terpenes in the emission blend for all species was limonene, the total enhancement in the emission at high carbon dioxide could be explained by increased activity of limonene synthase [52]. On the other hand, it has been demonstrated that isoprene emission decreases under high carbon dioxide due to either the stimulation of phosphoenolpyruvate carboxylase (PEPC), which competes for the pyruvate required for the MEP pathway, or the reduction in DMADP production [14]. However, in our experiment, the chlorophyll content at elevated carbon dioxide increases, suggesting that the N content in the leaves is not affected [53]. Indeed, the enhancement in soil-nitrogen supply affects the energy cycling between the reaction center and the chlorophyll pool, determining the chlorophyll increase. In contrast, the total phenolic compounds decrease for some species grown at elevated carbon dioxide due to the downregulation of the key enzyme PAL activity on the phenylpropanoid pathway [54]. Nonetheless, for most species, the total phenols are not significantly modified. The flavonoid contents decrease at elevated carbon dioxide for all species, probably due to a downregulation of leaf antioxidant enzymes under elevated carbon dioxide. The same trend has been found in the seedling of *Oryza sativa* L., while for mature plants, the total phenols and flavonoids concentration increase [55]. 

The hierarchical model of ANOVA analysis between treatment (different carbon dioxide concentrations) and species/varieties confirms the statistical significance of the differences between species and varieties within treatment (Table 2). 

Some structural changes could be seen for plants growing at high carbon dioxide. Such a modification in mitochondria and chloroplast has been shown for plants from different families and could be due to increased cellular energy demands when plants are grown at elevated CO_2_ [56]. 

## 4. Materials and Methods

### 4.1. Plant Material

The *Brassicaceae* (*Cruciferae* or mustard) family includes many species distributed worldwide (except Antarctica) and encloses approximately 338 genera and 3709 species.

Plants of 13 species from the *Brassicaceae* family were grown from the seeds as follows: Red cabbage (*Brassica oleracea* var. *capitata*, Langedijker Herfst (Sem-Luca, Timisoara, Romania)), Broccoli (*Brassica oleracea* var. *cymose*, Calabrese (Agrosel, Campia-Turzii, Romania)), Green Cabbage (*Brassica oleracea* var. *capitata*, Varza de buzau (Sem-Luca, Timisoara, Romania); Vertus 2, (Legutko, Jutrosin, Poland), Cuor de Bue Grosso, (Legutko, Jutrosin, Poland)), Kale (*Brassica oleracea* var. *sabellica*, Black magic (Sem-Luca, Timisoara, Romania)), Broccoli (*Brassica oleracea* var. *italica*, Early Purple (Legutko, Jutrosin, Poland)), Brussels sprout (*Brassica oleracea* var. *gemmifera*, Groninger (Sem-Luca, Timisoara, Romania)), Kohlrabi (*Brassica oleracea* var. *gongyloides*, Gongylodes (Agrosel, Campia-Turzii, Romania)), Cauliflower (*Brassica oleracea* var. *botrytis*, Moldovita F1 (Agrosel, Campia-Turzii, Romania)), Cress (*Lepidium sativum*, Common (Legutko, Jutrosin, Poland)), Rapeseed (*Brassica napus* subsp. *napus*, Pioneer PT275, Pioneer Hi-Bred, România), and White Mustard (*Sinapis alba* L., Franchi Sementini, Bergamo, Italy).

The seeds were sown in 0.8 L plastic pots filled with a mixture of commercial garden soil and quartz sand. The plants have been fertilized with fertilizers for foliar (Bionat Plus, Panetone SRL, Timisoara, Romania) and radicular (Cropcare 11-11-21, YaraMila, Oslo, Norway). Day length was 12 h, and the light intensity at plant level of 800 μmol m^−2^ s^−1^ was provided by led lamps (Hoff, Nürnberg, Germany). Day/night temperatures were maintained at 25/22 °C and a relative humidity of 65%. The plants were watered every day to soil field capacity. Seven-week-old non-bolted plants with at least three fully developed leaves were used in the experiments. We used the fully expanded leaves with the same development stage for all measurements. The plants were randomized to ensure that all plants grow in the same light. The leaves used for photosynthetic and volatile organic compounds measurements were used for the biochemical analysis. 

### 4.2. Photosynthetic Measurements

A portable gas exchange system (GFS-3000, Waltz, Effeltrich, Germany) was used to determine the photosynthetic parameters, as reported earlier in [57,58]. The calculation of the steady-state values of net assimilation (*A*) and stomatal conductance to water vapor (*g_s_*) was performed as was depicted in [58].

### 4.3. Volatile Sampling and GC–MS Analyses

Volatile organic compounds (VOC) were sampled (by a flow air sample pump 210-1003 MTX (SKC Inc., Houston, TX, USA)) and analyzed (by a Shimadzu TD20 automated cartridge desorber coupled with a Shimadzu 2010 Plus GC–MS equipment (Shimadzu Corporation, Kyoto, Japan)), as earlier reported in [59].

### 4.4. Chromatographic Analysis of Photosynthetic Pigments

The pigments (chlorophyll a, chlorophyll b, and β-carotene) were extracted in acetone as described before [60], and the quantitative analyses were performed using the UHPLC-DAD apparatus (NEXERA 8030, Shimadzu, Kyoto, Japan) following the same method earlier published [57]. The concentration of chlorophyll a, chlorophyll b, and β-carotene was calculated using the pure chromatographic standards (Merck, Darmstadt, Germany).

### 4.5. Flavonoid Content Analysis

The total flavonoid content was determined using the spectrophotometric method [59]. A reaction mixture of aluminum chloride, sodium acetate, and sample was measured at 434 nm, and the results were expressed in mg rutin equivalents/mL. 

### 4.6. Total Phenolic Content—Folin–Ciocalteu Method

Total phenolic content was determined according to the Folin–Ciocalteu, method as described in [59,61], and the results were expressed in mg gallic acid equivalents/mL.

### 4.7. Microscopy Analyses

The surfaces of leaves were examined by using a Zeiss Scope.A1 microscope equipped with the AxioCam MRc 5 camera and ZEN lite 2012 software (Carl Zeiss MicroImaging GmbH, Jena, Germany). The samples preparation was done by following the next steps: peel the epidermis from the backside of the leaf, mount the sample on a glass slide in distilled water, fix with a coverslip, and observe under the microscope at 40× magnification. For scanning electron microscopy (SEM), leaves were mounted on stubs using carbon double-sided adhesive tape without any treatment. The samples were examined and photographed using LYRA3 scanning electron microscope (LYRA3 XMU, Tescan, Brno, CzechRepublic) with Low Vacuum Secondary Electron Tescan Detector (LVSTD), at 15 kV and magnification 800×. 

### 4.8. Statistical Analysis and Data Handling

One-way ANOVA, Tukey’s multiple comparisons test, and two-way ANOVA were done using GraphPad Prism version 9.3.0 for Windows (GraphPad Software, San Diego, Ca, USA, www.graphpad.com (accessed on 30 January 2022)). Results were considered significantly different at *p*-values < 0.05.

## 5. Conclusions

In this study, we have shown that elevated carbon dioxide increases the photosynthetic activities and emission of volatile organic compounds. On the other hand, plants that grow at a high concentration of CO_2_ exhibit downregulation of polyphenols and flavonoids, which could become a significant problem in light of future climate change conditions. The results revealed that different species/varieties from the *Brassicaceae* family respond differently to increases in carbon dioxide concentration. Generally, all plants increase their assimilation rates, monoterpene emission, and chlorophylls and decrease their flavonoids and polyphenols content. 

## Figures and Tables

**Table 1:** Examples of the effect of increased CO_2_ concentrations on the photosynthetic characteristics, secondary metabolites, and pigment content of different plant species.

Plant Characteristic	Effect *	Species	Elevated Carbon Dioxide Concentration (ppmv)	Reference
Assimilation rate	↑	*Trifolium repens*	600	[9]
	↑	*Triticum aestivum*	583	[7]
	↑	*Oryza sativa*	475–600	[29]
	↑	*Glycine max*	475–600	[29]
	↑	*Carthamus tinctorius*	1000	[30]
	↑	*Phaseolus vulgaris* L.	700	[31]
	↑	*Populus* × *euroamericana*	550	[32]
	↑	*Camellia sinensis*	770	[17]
	↑	*Oryza sativa* L.	570	[20]
Isoprene emission	↓	*Phragmites australis*	800	[14]
	↓	*Platanus* × *acerfolia*	800	[14]
	↓	*Populas nigra* × *maximowiczii NM6*	800	[14]
	↓	*Populus* × *euroamericana*	550	[32]
	↔	*Populus tremula* × *Populus tremuloides*	780	[33]
Total phenols	↑	*Camellia sinensis*	770	[17]
	↑	*Cicer arietinum*	750	[18]
	↑	*Baccharis dracunculifolia*	750–800	[19]
	↑	*Baccharis platypoda*	750–800	[19]
Chlorophylls concentration	↓	*Oryza sativa* L.	570	[20]
	↓	*Solanum lycopersicum*	1000	[21]
	↑	*Cicer arietinum*	700	[23]
	↑	*Brassica rapa*	800	[24]
	↑	*Sorghum bicolor*	700	[25]
	↔	*Carthamus tinctorius*	1000	[30]
Total flavonoids	↑	*Robinia pseudoacacia* L.	750	[26]
	↓	*Hordeum vulgare*	620	[28]
	↓	*Zea maize*	620	[28]

* Effect: unchanged: ↔; increase: ↑; decrease: ↓.

**Table 2:** The hierarchical model of ANOVA analysis between treatment (different carbon dioxide concentrations) and species/varieties.

Source of Variation	Assimilation Rate	Stomata Conductance	Monoterpene Emission	Chlorophyll *a*	Chlorophyll *b*	β-carotene	Flavonoids	Polyphenols
	df, MS, F, *p* Value	df, MS, F, *p* Value	df, MS, F, *p* Value	df, MS, F, *p* Value	df, MS, F, *p* Value	df, MS, F, *p* Value	df, MS, F, *p* Value	df, MS, F, *p* Value
Carbon dioxide	2, 3182, 2849, <0.0001	2, 4907, 97.81, <0.0001	2, 0.0406, 1671, <0.0001	2, 119,198, 214.2, <0.0001	2, 39,098, 130.2, <0.0001	2, 1127, 44.26, <0.0001	2, 0.00333, 265.4, <0.0001	2, 2573, 5178, <0.0001
Species/varieties	12, 313.2, 280.4, <0.0001	12, 7206, 143.6, <0.0001	12, 0.0724, 2979, <0.0001	12, 25,178, 45.24, <0.0001	12, 10,211, 34.01, <0.0001	12, 389.9, 15.31, <0.0001	12, 0.00629, 501.0, <0.0001	12, 22,126, 44,530, <0.0001
Error	174.2	7827	0.001895	43,406	23,416	1986	0.001958	38.76

## Data Availability

The data presented in this study are available in the article.
